# 2-{(1S*,2S*)-2-[(*E*)-(2,4-Dihy­droxy­benzyl­idene)amino]­cyclo­hex­yl}isoindoline-1,3-dione

**DOI:** 10.1107/S1600536811019787

**Published:** 2011-06-04

**Authors:** Zhi-Jian Liu, Xiang-Kai Fu, Zhong-Kai Hu, Xiao-Ju Wu, Liu Wu

**Affiliations:** a College of Chemistry and Chemical Engineering, Research Institute of Applied Chemistry, Southwest University, The Key Laboratory of Applied Chemistry of Chongqing Municipality, Chongqing 400715, People’s Republic of China

## Abstract

In the title mol­ecule, C_21_H_20_N_2_O_4_, the dihedral angle between the phenol ring and the isoindole-1,3-dione mean plane is 69.79 (6)°. The cyclo­hexane ring adopts a chair conformation. Weak inter­molecular O—H⋯O and O—H⋯N inter­actions feature as part of the crystal packing.

## Related literature

For details of the synthesis, see: Berkessel *et al.* (2006 ▶); Ren & Fu (2009 ▶). For background to the synthesis of salen-type Schiff base ligands, see: Campbell & Nguyen (2001 ▶).
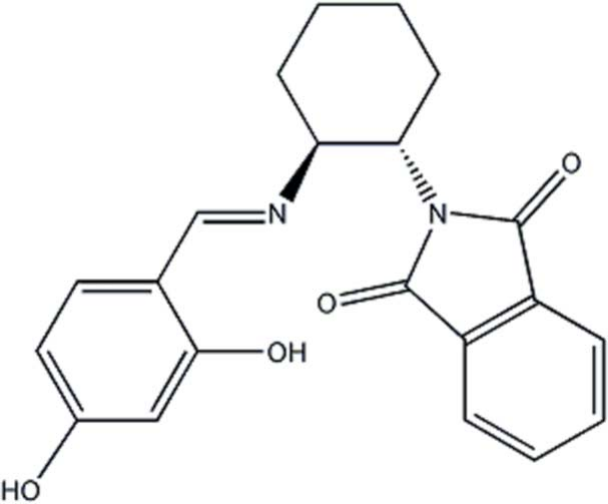


## Experimental

### 

#### Crystal data

C_21_H_20_N_2_O_4_
*M* *_r_* = 364.39
Orthorhombic, 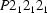

*a* = 9.0247 (3) Å
*b* = 11.7748 (4) Å
*c* = 17.8585 (6) Å
*V* = 1897.72 (11) Å^3^
*Z* = 4
Mo *K*α radiation
μ = 0.09 mm^−1^
*T* = 296 K
0.20 × 0.20 × 0.20 mm

#### Data collection

Bruker APEX CCD area-detector diffractometer
Absorption correction: multi-scan (*SADABS*; Sheldrick, 1996 ▶) *T*_min_ = 0.982, *T*_max_ = 0.982
34622 measured reflections
4727 independent reflections
4317 reflections with *I* > 2σ(*I*)
*R*_int_ = 0.023

#### Refinement

*R*[*F* ^2^ > 2σ(*F* ^2^)] = 0.041
*wR*(*F* ^2^) = 0.132
*S* = 1.10
4727 reflections
251 parameters
1 restraint
H atoms treated by a mixture of independent and constrained refinement
Δρ_max_ = 0.26 e Å^−3^
Δρ_min_ = −0.32 e Å^−3^

### 

Data collection: *SMART* (Bruker, 2001 ▶); cell refinement: *SAINT-Plus* (Bruker, 2001 ▶); data reduction: *SAINT-Plus*; program(s) used to solve structure: *SHELXS97* (Sheldrick, 2008 ▶); program(s) used to refine structure: *SHELXL97* (Sheldrick, 2008 ▶); molecular graphics: *SHELXTL* (Sheldrick, 2008 ▶); software used to prepare material for publication: *SHELXTL*.

## Supplementary Material

Crystal structure: contains datablock(s) I, global. DOI: 10.1107/S1600536811019787/ff2012sup1.cif

Structure factors: contains datablock(s) I. DOI: 10.1107/S1600536811019787/ff2012Isup2.hkl

Supplementary material file. DOI: 10.1107/S1600536811019787/ff2012Isup3.cml

Additional supplementary materials:  crystallographic information; 3D view; checkCIF report

## Figures and Tables

**Table 1:** Hydrogen-bond geometry (Å, °)

*D*—H⋯*A*	*D*—H	H⋯*A*	*D*⋯*A*	*D*—H⋯*A*
O3—H3*A*⋯N2	0.93 (1)	1.69 (1)	2.5656 (16)	157 (2)
O4—H4*B*⋯O3^i^	0.90 (2)	1.66 (2)	2.5478 (15)	170 (2)

Symmetry code: (i) 
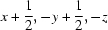
.

**Fig. 1.**
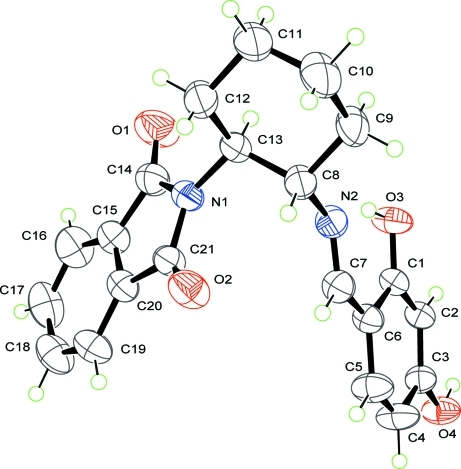
Molecular structure showing 30% probability displacement ellipsoids.

## supplementary crystallographic information

### Comment

Salen-type Schiff base ligands incorporating two different benzylidene moieties and a diamine backbone were synthesized in high yield under mild conditions *via* a stepwise approach. In the synthesis of salen-type Schiff base ligands (Campbell *et al.*, 2001), the compound (I) was a significant intermediate product. Here we report its crystal structure.

### Experimental

Compound (I) was synthesized according to the procedure of Berkessel *et al.* (2006); Ren *et al.* (2009). A crystal of (I) suitable for X-ray analysis was grown from a mixture solution of ethanol and acetonitrile (1:1) by slow evaporation at room temperature.

### Refinement

All the hydrogen atoms were placed at the geometrical positions with C—H = 0.93 Å(CH), 0.97Å (CH), 0.97Å (CH_2_), and refined as riding, with *U*_iso_(H) = 1.2 *U*_eq_(C) and *U*_iso_(H) = 1~1.4 *U*_eq_(O). A restrained refinement comment "*DFIX*" is used to restraint the distance of O3 and H3a.

### Crystal data

**Table:**

C_21_H_20_N_2_O_4_	*D*_x_ = 1.275 Mg m^−^^3^
*M**_r_* = 364.39	Mo *K*α radiation, λ = 0.71073 Å
Orthorhombic, *P*2_1_2_1_2_1_	Cell parameters from 34622 reflections
*a* = 9.0247 (3) Å	θ = 2.1–28.3°
*b* = 11.7748 (4) Å	µ = 0.09 mm^−^^1^
*c* = 17.8585 (6) Å	*T* = 296 K
*V* = 1897.72 (11) Å^3^	Block, colourless
*Z* = 4	0.20 × 0.20 × 0.20 mm
*F*(000) = 768	

### Data collection

**Table:**

Bruker APEX CCD area-detector diffractometer	4727 independent reflections
Radiation source: fine-focus sealed tube	4317 reflections with *I* > 2σ(*I*)
graphite	*R*_int_ = 0.023
φ and ω scans	θ_max_ = 28.3°, θ_min_ = 2.1°
Absorption correction: multi-scan (*SADABS*; Sheldrick, 1996)	*h* = −12→12
*T*_min_ = 0.982, *T*_max_ = 0.982	*k* = −15→15
34622 measured reflections	*l* = −23→23

### Refinement

**Table:**

Refinement on *F*^2^	Primary atom site location: structure-invariant direct methods
Least-squares matrix: full	Secondary atom site location: difference Fourier map
*R*[*F*^2^ > 2σ(*F*^2^)] = 0.041	Hydrogen site location: inferred from neighbouring sites
*wR*(*F*^2^) = 0.132	H atoms treated by a mixture of independent and constrained refinement
*S* = 1.10	*w* = 1/[σ^2^(*F*_o_^2^) + (0.1*P*)^2^] where *P* = (*F*_o_^2^ + 2*F*_c_^2^)/3
4727 reflections	(Δ/σ)_max_ < 0.001
251 parameters	Δρ_max_ = 0.26 e Å^−^^3^
1 restraint	Δρ_min_ = −0.32 e Å^−^^3^

### Special details

**Table:**

Geometry. All e.s.d.'s (except the e.s.d. in the dihedral angle between two l.s. planes) are estimated using the full covariance matrix. The cell e.s.d.'s are taken into account individually in the estimation of e.s.d.'s in distances, angles and torsion angles; correlations between e.s.d.'s in cell parameters are only used when they are defined by crystal symmetry. An approximate (isotropic) treatment of cell e.s.d.'s is used for estimating e.s.d.'s involving l.s. planes.
Refinement. Refinement of *F*^2^ against ALL reflections. The weighted *R*-factor *wR* and goodness of fit *S* are based on *F*^2^, conventional *R*-factors *R* are based on *F*, with *F* set to zero for negative *F*^2^. The threshold expression of *F*^2^ > σ(*F*^2^) is used only for calculating *R*-factors(gt) *etc*. and is not relevant to the choice of reflections for refinement. *R*-factors based on *F*^2^ are statistically about twice as large as those based on *F*, and *R*- factors based on ALL data will be even larger.

### Fractional atomic coordinates and isotropic or equivalent isotropic displacement parameters (Å^2^)

**Table:**

	*x*	*y*	*z*	*U*_iso_*/*U*_eq_	
C1	0.30161 (15)	0.36416 (11)	0.06573 (7)	0.0377 (3)	
C2	0.42917 (15)	0.34393 (11)	0.02192 (7)	0.0388 (3)	
H2B	0.4445	0.2721	0.0017	0.047*	
C3	0.53102 (15)	0.42835 (11)	0.00866 (7)	0.0405 (3)	
C4	0.5076 (2)	0.53951 (12)	0.03634 (11)	0.0576 (4)	
H4A	0.5759	0.5967	0.0262	0.069*	
C5	0.3856 (2)	0.56221 (12)	0.07755 (10)	0.0551 (4)	
H5A	0.3702	0.6358	0.0948	0.066*	
C6	0.28074 (16)	0.47702 (11)	0.09522 (7)	0.0400 (3)	
C7	0.15624 (16)	0.50289 (12)	0.13977 (7)	0.0442 (3)	
H7A	0.1438	0.5774	0.1559	0.053*	
C8	−0.07381 (16)	0.45130 (14)	0.20425 (7)	0.0452 (3)	
H8A	−0.0812	0.5330	0.2141	0.054*	
C9	−0.21031 (18)	0.4117 (2)	0.16090 (8)	0.0604 (4)	
H9A	−0.2190	0.4554	0.1151	0.072*	
H9B	−0.1986	0.3324	0.1474	0.072*	
C10	−0.3512 (2)	0.4259 (3)	0.20717 (11)	0.0762 (6)	
H10A	−0.4352	0.3973	0.1790	0.091*	
H10B	−0.3678	0.5060	0.2169	0.091*	
C11	−0.3396 (2)	0.3625 (3)	0.28091 (10)	0.0809 (7)	
H11A	−0.4287	0.3751	0.3102	0.097*	
H11B	−0.3311	0.2817	0.2713	0.097*	
C12	−0.2046 (2)	0.4032 (2)	0.32491 (9)	0.0683 (5)	
H12A	−0.1969	0.3608	0.3713	0.082*	
H12B	−0.2160	0.4829	0.3372	0.082*	
C13	−0.06454 (17)	0.38642 (13)	0.27883 (7)	0.0458 (3)	
H13A	−0.0588	0.3054	0.2665	0.055*	
C14	0.1428 (2)	0.33206 (13)	0.36431 (9)	0.0524 (4)	
C15	0.27356 (18)	0.38879 (13)	0.39836 (8)	0.0480 (3)	
C16	0.3843 (2)	0.34711 (16)	0.44396 (10)	0.0667 (5)	
H16A	0.3845	0.2716	0.4592	0.080*	
C17	0.4951 (2)	0.4208 (2)	0.46639 (11)	0.0683 (5)	
H17A	0.5710	0.3945	0.4971	0.082*	
C18	0.4943 (2)	0.53120 (17)	0.44398 (10)	0.0631 (5)	
H18A	0.5714	0.5787	0.4585	0.076*	
C19	0.38096 (18)	0.57462 (14)	0.39993 (10)	0.0549 (4)	
H19A	0.3792	0.6507	0.3861	0.066*	
C20	0.27078 (16)	0.50062 (12)	0.37739 (7)	0.0432 (3)	
C21	0.13765 (16)	0.52076 (11)	0.33052 (7)	0.0420 (3)	
N1	0.06951 (15)	0.41440 (10)	0.32197 (6)	0.0446 (3)	
N2	0.05849 (14)	0.42829 (11)	0.15940 (6)	0.0445 (3)	
O1	0.1041 (2)	0.23425 (11)	0.36929 (9)	0.0804 (5)	
O2	0.09328 (16)	0.60904 (9)	0.30445 (7)	0.0612 (3)	
O3	0.20554 (13)	0.28623 (9)	0.07975 (7)	0.0560 (3)	
H3A	0.141 (2)	0.3207 (16)	0.1129 (9)	0.065*	
O4	0.65643 (13)	0.41288 (9)	−0.02971 (7)	0.0526 (3)	
H4B	0.665 (3)	0.340 (2)	−0.0447 (12)	0.070 (6)*	

### Atomic displacement parameters (Å^2^)

**Table:**

	*U*^11^	*U*^22^	*U*^33^	*U*^12^	*U*^13^	*U*^23^
C1	0.0332 (6)	0.0376 (6)	0.0423 (5)	−0.0025 (5)	−0.0030 (5)	−0.0034 (4)
C2	0.0352 (6)	0.0353 (5)	0.0459 (5)	−0.0004 (5)	−0.0008 (5)	−0.0045 (5)
C3	0.0371 (6)	0.0386 (6)	0.0459 (6)	0.0001 (5)	0.0037 (5)	−0.0001 (5)
C4	0.0567 (9)	0.0350 (6)	0.0812 (10)	−0.0097 (6)	0.0230 (8)	−0.0054 (6)
C5	0.0574 (9)	0.0341 (6)	0.0737 (9)	−0.0044 (6)	0.0188 (8)	−0.0091 (6)
C6	0.0361 (6)	0.0399 (6)	0.0439 (5)	−0.0015 (5)	0.0010 (5)	−0.0044 (5)
C7	0.0423 (7)	0.0450 (6)	0.0452 (6)	0.0012 (6)	0.0027 (5)	−0.0045 (5)
C8	0.0337 (6)	0.0610 (8)	0.0408 (5)	−0.0011 (6)	0.0023 (5)	−0.0061 (5)
C9	0.0374 (7)	0.0999 (13)	0.0439 (6)	−0.0054 (9)	−0.0031 (6)	−0.0076 (7)
C10	0.0341 (8)	0.130 (2)	0.0648 (9)	−0.0055 (11)	−0.0019 (7)	−0.0189 (11)
C11	0.0448 (9)	0.141 (2)	0.0570 (9)	−0.0316 (12)	0.0100 (7)	−0.0177 (10)
C12	0.0469 (9)	0.1134 (16)	0.0445 (7)	−0.0239 (10)	0.0069 (7)	−0.0135 (8)
C13	0.0407 (7)	0.0544 (7)	0.0423 (6)	−0.0122 (6)	−0.0009 (5)	−0.0059 (5)
C14	0.0586 (9)	0.0461 (7)	0.0524 (7)	−0.0066 (7)	−0.0056 (7)	0.0016 (6)
C15	0.0469 (8)	0.0470 (7)	0.0502 (6)	0.0010 (6)	−0.0045 (6)	−0.0030 (5)
C16	0.0713 (12)	0.0577 (9)	0.0712 (10)	0.0147 (9)	−0.0154 (9)	0.0034 (8)
C17	0.0510 (9)	0.0877 (13)	0.0661 (9)	0.0242 (9)	−0.0171 (8)	−0.0182 (9)
C18	0.0387 (8)	0.0766 (11)	0.0741 (10)	0.0040 (8)	−0.0097 (7)	−0.0236 (9)
C19	0.0422 (8)	0.0529 (8)	0.0695 (9)	−0.0048 (7)	−0.0075 (7)	−0.0107 (7)
C20	0.0391 (7)	0.0449 (6)	0.0456 (6)	0.0002 (6)	−0.0024 (5)	−0.0063 (5)
C21	0.0405 (7)	0.0408 (6)	0.0445 (6)	−0.0048 (5)	−0.0035 (5)	−0.0025 (5)
N1	0.0430 (6)	0.0445 (6)	0.0462 (5)	−0.0079 (5)	−0.0059 (5)	−0.0017 (4)
N2	0.0368 (6)	0.0543 (6)	0.0425 (5)	−0.0017 (5)	0.0042 (4)	−0.0061 (4)
O1	0.1011 (12)	0.0451 (6)	0.0951 (9)	−0.0188 (7)	−0.0246 (9)	0.0109 (6)
O2	0.0689 (8)	0.0429 (5)	0.0719 (6)	−0.0015 (5)	−0.0229 (6)	0.0039 (5)
O3	0.0460 (6)	0.0466 (5)	0.0756 (7)	−0.0154 (5)	0.0170 (5)	−0.0165 (5)
O4	0.0426 (6)	0.0449 (5)	0.0703 (6)	−0.0043 (4)	0.0187 (5)	−0.0051 (5)

### Geometric parameters (Å, °)

**Table:**

C1—O3	1.2869 (16)	C11—H11A	0.9700
C1—C2	1.4122 (18)	C11—H11B	0.9700
C1—C6	1.4418 (17)	C12—C13	1.521 (2)
C2—C3	1.3744 (19)	C12—H12A	0.9700
C2—H2B	0.9300	C12—H12B	0.9700
C3—O4	1.3355 (16)	C13—N1	1.4716 (17)
C3—C4	1.4150 (19)	C13—H13A	0.9800
C4—C5	1.351 (2)	C14—O1	1.207 (2)
C4—H4A	0.9300	C14—N1	1.396 (2)
C5—C6	1.415 (2)	C14—C15	1.486 (2)
C5—H5A	0.9300	C15—C20	1.369 (2)
C6—C7	1.4101 (19)	C15—C16	1.380 (2)
C7—N2	1.2933 (19)	C16—C17	1.383 (3)
C7—H7A	0.9300	C16—H16A	0.9300
C8—N2	1.4630 (17)	C17—C18	1.361 (3)
C8—C9	1.528 (2)	C17—H17A	0.9300
C8—C13	1.5377 (19)	C18—C19	1.388 (2)
C8—H8A	0.9800	C18—H18A	0.9300
C9—C10	1.525 (2)	C19—C20	1.382 (2)
C9—H9A	0.9700	C19—H19A	0.9300
C9—H9B	0.9700	C20—C21	1.4833 (19)
C10—C11	1.518 (3)	C21—O2	1.2073 (18)
C10—H10A	0.9700	C21—N1	1.4036 (18)
C10—H10B	0.9700	O3—H3A	0.927 (9)
C11—C12	1.527 (2)	O4—H4B	0.90 (2)
			
O3—C1—C2	122.46 (11)	H11A—C11—H11B	108.1
O3—C1—C6	119.88 (12)	C11—C12—C13	110.13 (13)
C2—C1—C6	117.66 (11)	C11—C12—H12A	109.6
C3—C2—C1	121.25 (11)	C13—C12—H12A	109.6
C3—C2—H2B	119.4	C11—C12—H12B	109.6
C1—C2—H2B	119.4	C13—C12—H12B	109.6
O4—C3—C2	123.82 (12)	H12A—C12—H12B	108.1
O4—C3—C4	115.58 (12)	N1—C13—C12	111.77 (10)
C2—C3—C4	120.60 (12)	N1—C13—C8	112.77 (11)
C5—C4—C3	119.66 (13)	C12—C13—C8	111.04 (14)
C5—C4—H4A	120.2	N1—C13—H13A	107.0
C3—C4—H4A	120.2	C12—C13—H13A	107.0
C4—C5—C6	121.77 (13)	C8—C13—H13A	107.0
C4—C5—H5A	119.1	O1—C14—N1	124.41 (16)
C6—C5—H5A	119.1	O1—C14—C15	128.97 (17)
C7—C6—C5	120.39 (12)	N1—C14—C15	106.61 (12)
C7—C6—C1	120.61 (12)	C20—C15—C16	121.12 (15)
C5—C6—C1	118.98 (12)	C20—C15—C14	107.81 (13)
N2—C7—C6	123.35 (13)	C16—C15—C14	131.07 (15)
N2—C7—H7A	118.3	C17—C16—C15	118.15 (17)
C6—C7—H7A	118.3	C17—C16—H16A	120.9
N2—C8—C9	108.92 (11)	C15—C16—H16A	120.9
N2—C8—C13	109.74 (12)	C18—C17—C16	120.68 (17)
C9—C8—C13	109.34 (13)	C18—C17—H17A	119.7
N2—C8—H8A	109.6	C16—C17—H17A	119.7
C9—C8—H8A	109.6	C17—C18—C19	121.53 (17)
C13—C8—H8A	109.6	C17—C18—H18A	119.2
C10—C9—C8	111.36 (12)	C19—C18—H18A	119.2
C10—C9—H9A	109.4	C20—C19—C18	117.58 (16)
C8—C9—H9A	109.4	C20—C19—H19A	121.2
C10—C9—H9B	109.4	C18—C19—H19A	121.2
C8—C9—H9B	109.4	C15—C20—C19	120.90 (14)
H9A—C9—H9B	108.0	C15—C20—C21	108.84 (13)
C9—C10—C11	111.02 (17)	C19—C20—C21	130.27 (14)
C9—C10—H10A	109.4	O2—C21—N1	125.52 (13)
C11—C10—H10A	109.4	O2—C21—C20	128.60 (13)
C9—C10—H10B	109.4	N1—C21—C20	105.88 (11)
C11—C10—H10B	109.4	C14—N1—C21	110.67 (11)
H10A—C10—H10B	108.0	C14—N1—C13	121.18 (12)
C12—C11—C10	110.31 (18)	C21—N1—C13	128.09 (12)
C12—C11—H11A	109.6	C7—N2—C8	125.40 (13)
C10—C11—H11A	109.6	C1—O3—H3A	103.8 (13)
C12—C11—H11B	109.6	C3—O4—H4B	110.7 (15)
C10—C11—H11B	109.6		
			
O3—C1—C2—C3	−179.12 (13)	C20—C15—C16—C17	−1.7 (3)
C6—C1—C2—C3	0.89 (18)	C14—C15—C16—C17	178.88 (18)
C1—C2—C3—O4	177.16 (12)	C15—C16—C17—C18	0.0 (3)
C1—C2—C3—C4	−2.5 (2)	C16—C17—C18—C19	1.9 (3)
O4—C3—C4—C5	−178.20 (16)	C17—C18—C19—C20	−2.1 (3)
C2—C3—C4—C5	1.5 (3)	C16—C15—C20—C19	1.5 (2)
C3—C4—C5—C6	1.1 (3)	C14—C15—C20—C19	−178.98 (14)
C4—C5—C6—C7	178.57 (16)	C16—C15—C20—C21	−178.48 (14)
C4—C5—C6—C1	−2.7 (3)	C14—C15—C20—C21	1.04 (17)
O3—C1—C6—C7	0.4 (2)	C18—C19—C20—C15	0.4 (2)
C2—C1—C6—C7	−179.62 (11)	C18—C19—C20—C21	−179.62 (14)
O3—C1—C6—C5	−178.38 (14)	C15—C20—C21—O2	176.27 (16)
C2—C1—C6—C5	1.6 (2)	C19—C20—C21—O2	−3.7 (3)
C5—C6—C7—N2	−178.23 (14)	C15—C20—C21—N1	−3.42 (16)
C1—C6—C7—N2	3.0 (2)	C19—C20—C21—N1	176.60 (15)
N2—C8—C9—C10	176.03 (17)	O1—C14—N1—C21	177.16 (18)
C13—C8—C9—C10	56.1 (2)	C15—C14—N1—C21	−4.00 (17)
C8—C9—C10—C11	−56.8 (2)	O1—C14—N1—C13	−0.2 (3)
C9—C10—C11—C12	57.0 (3)	C15—C14—N1—C13	178.65 (12)
C10—C11—C12—C13	−57.9 (3)	O2—C21—N1—C14	−175.12 (16)
C11—C12—C13—N1	−174.55 (17)	C20—C21—N1—C14	4.58 (16)
C11—C12—C13—C8	58.6 (2)	O2—C21—N1—C13	2.0 (2)
N2—C8—C13—N1	56.93 (16)	C20—C21—N1—C13	−178.30 (12)
C9—C8—C13—N1	176.34 (13)	C12—C13—N1—C14	89.91 (19)
N2—C8—C13—C12	−176.74 (13)	C8—C13—N1—C14	−144.14 (14)
C9—C8—C13—C12	−57.33 (18)	C12—C13—N1—C21	−86.94 (19)
O1—C14—C15—C20	−179.50 (19)	C8—C13—N1—C21	39.01 (19)
N1—C14—C15—C20	1.73 (18)	C6—C7—N2—C8	−178.75 (12)
O1—C14—C15—C16	0.0 (3)	C9—C8—N2—C7	124.08 (16)
N1—C14—C15—C16	−178.81 (17)	C13—C8—N2—C7	−116.25 (15)

### Hydrogen-bond geometry (Å, °)

**Table:**

*D*—H···*A*	*D*—H	H···*A*	*D*···*A*	*D*—H···*A*
O3—H3A···N2	0.93 (1)	1.69 (1)	2.5656 (16)	157.(2)
O4—H4B···O3^i^	0.90 (2)	1.66 (2)	2.5478 (15)	170 (2)

Symmetry codes: (i) *x*+1/2, −*y*+1/2, −*z*.
